# Oral manifestations in drug users: A review

**DOI:** 10.4317/jced.55928

**Published:** 2020-02-01

**Authors:** Federico Cossa, Alessia Piastra, Mª Gracia Sarrion-Pérez, Leticia Bagán

**Affiliations:** 1 Student of the master of Implantology at the Universidad Europea de Valencia. Graduated in Dentistry at the Universidad Europea de Valencia; 2 Student of the master of Endodontics at the University of Valencia. Graduated in Dentistry at the Universidad Europea de Valencia; 3 PhD, Associate Professor. Faculty of Health Sciences. Department of Dentistry. European University of Valencia. Spain; 4 PhD, Titular professor. Faculty of Health Sciences. Department of Dentistry. European University of Valencia. Spain

## Abstract

**Background:**

In the dental environment there is not much talk about the oral manifestations resulting from the use of drugs, because in general the issue of drugs is a very difficult subject to deal with. Therefore, the objective of this work is to understand what are the most obvious manifestations in the oral cavity and as the dentist can detect them.

**Material and Methods:**

In order to carry out this bibliographical review, a scientific article search was made by consulting the PubMed database. The abstracts were read to recruit only what was consistent with the chosen topic.

**Results:**

The 23 sources of information affirmed the relationship between a bad state of general health, and more in the specific, bad state of oral cavity.

**Conclusions:**

The most relevant manifestations were: decay, loss of teeth and precancerous lesions. These manifestations were present in most of the cases studied. All this is a consequence of the drug and the lifestyle acquired by the drug users studied.

** Key words:**Drug, caries, meth mouth, periodontitis, xerostomia.

## Introduction

In 1992, the WHO defined drug addiction as “a state, psychic and sometimes also physical, resulting in the interaction between a living organism and a drug, characterized by behavioral and other responses that always include a compulsion to take the drug on a continuous or periodic basis in order to experience its psychic effects, and sometimes to avoid the discomfort of its absence. Tolerance may or may not be present (1).”

To date, drug abuse is one of the world’s most devastating health problems, the root cause of risky behaviors, violence, and social problems (2,3).

Globally, it is estimated that a 275 million of the adult population aged 15-64 years used drugs at least once in 2016. Some 31 million of substance abusers, are estimated to suffer from drug use disorders. Opiates, such as heroine and opium, are the substances that cause the highest negative health impact; however, cannabis remains the world’s most widely used drug, by an estimated 192.2 million people, amphetamines are still in second place with an estimated 34.2 million users, cocaine users amount to nearly 18.2 million worldwide, and approximately 19.4 million people have used opiates.

Estimated Figures of drug use in Europe in 2017 show 24 million adults aged 15-64 years used cannabis, 3.5 million cocaine users, an estimated 1.7 million amphetamine users and 1.3 million are estimated to have used opiates.

Many drug users tend to be polydrug users, making the entire drug-use scenario rather complicated.

The most common method of using cannabis is by smoking it mixed with tobacco; health problems may increase with the use of higher potency cannabis products, especially those with high concentrations of the psychoactive component, tetrahydrocannabinol. Common physical health problems include chronic respiratory symptoms or mental health problems, such as cannabis dependence and psychotic symptoms.

Snorting cocaine is common, however, marginalized users are more likely to inject it or smoke crack cocaine (4). Cocaine stimulates the dopamine system, inducing a feeling of euphoria and arousal (5), the most severe complications are seizures, hemorrhagic and ischemic strokes, myocardial infarction, aortic dissection, rhabdomyolysis, mesenteric ischemia, acute renal injury, multiple organ failure, and fast destructive processes in the midfacial region, and is important the differential diagnosis with these other diseases like Wegener´s granulomatosis, NK/T cell lymphoma, infections and other neoplasias (4,6). Cocaine use during pregnancy increases the risk of a foetus with a cleft palate (5).

Amphetamines may be inhaled or injected, smoked, swallowed as a pill or dissolved in a drink (7). These substances produce an intense rush of pleasure, a sense of euphoria and a general state of dehydration (8,9). People who inject drugs (PWID) are more exposed to infectious diseases such as HIV, hepatitis and tuberculosis, accidental overdose, and anesthesia complications (10).

Drug abuse has both direct and indirect consequences for oral health; the associated complications may result from direct exposure of oral tissues to drugs during smoking or ingestion, biologic interaction of drugs with normal physiology of oral cavity, and the effects of drugs on the brain and on systemic functions (2).

The aim of this bibliographic review is to explore the lesions that may appear in the oral area as a result of substance abuse, focusing on the most commonly used drugs nowadays.

## Material and Methods

In this bibliographic review research was carried out on PubMed for articles published from 2006 to 2018, the keywords being: “drug abuse”, “side effects”, “palatal perforation”, “oral health”, “dry mouth”, “caries”, “cocaine”, “heroine”, “methamphetamine”, “cannabis”, “marihuana”, “periodontal disease” and “dental disease”. These keywords were combined with the boolean operators “AND” and “OR”.

Review articles, clinical trials, comparative studies and cases series were included, as well as systematic reviews and metanalysis.

With the words we have chosen and the filters, we have found 194 articles. Of these, we have eliminated they also included drugs such as alcohol, medication, and tobacco.

**Figure 1:**
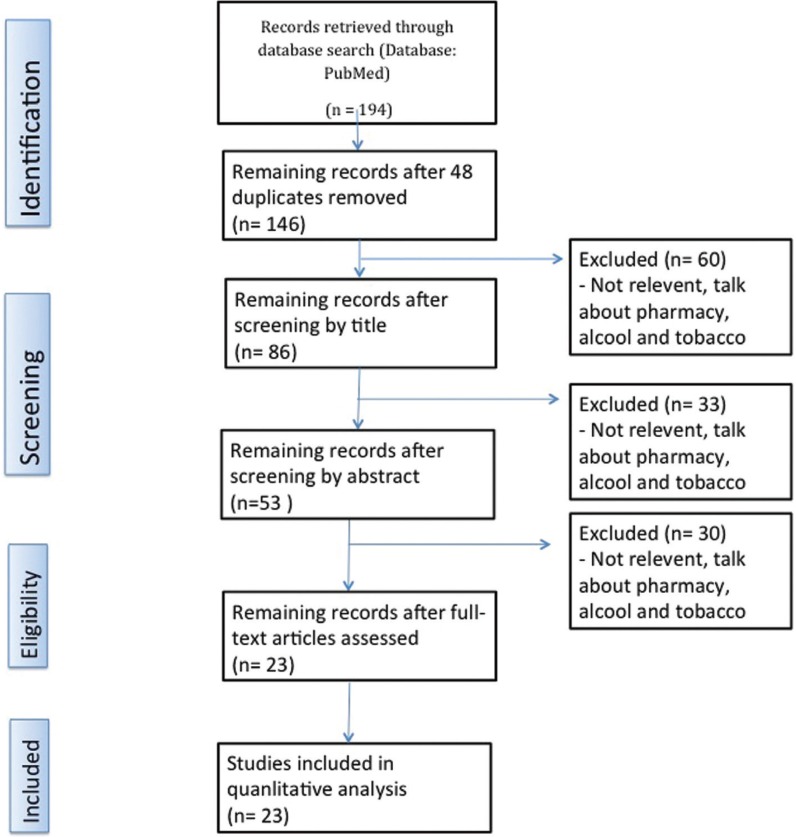
Flow diagram.

We drew on information from 23 articles to complete our work, in addition to 4 booklets by UNODC (United Nations Office on Drugs and Crime) and EMCDDA (European Monitoring Centre for Drugs and Drug Addiction). The selection process is illustrated as a PRISMA flow diagram, (Fig. 1).

## Results

Of the 23 articles, 3 were used only for introduction and were not included for the results, for the results of this review we have selected 20, of which: 2 retrospective cohort study, 3 reviews, 8 cross-sectional study, 1 prospective cohort study, 1 retrospective observational case, 1 systematic review, 3 pilot studies and 1 comparative study.

Of these articles, 3 speak only of cocaine, 1 speaks only of heroin, 1 of cannabis alone and 9 of methamphetamine alone. The remaining articles have been divided in mixed drugs: 3 articles bring together heroin, cocaine and cannabis; 2 articles put together cocaine and cannabis; 1 articles talk about heroin and methamphetamine.

The main characteristics of the different articles are detailed in  Table 1,  Table 1 cont.,  Table 1 cont.1.

**Table 1:**
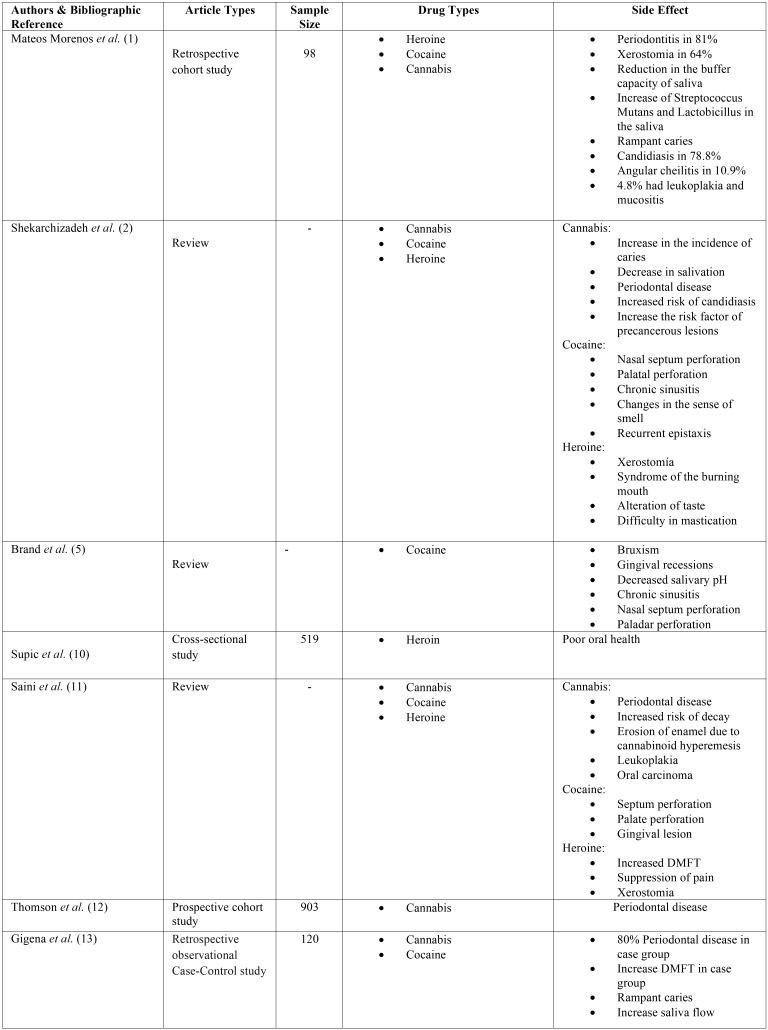
Description of the main characteristics of the selected studies.

**Table 1 cont.**
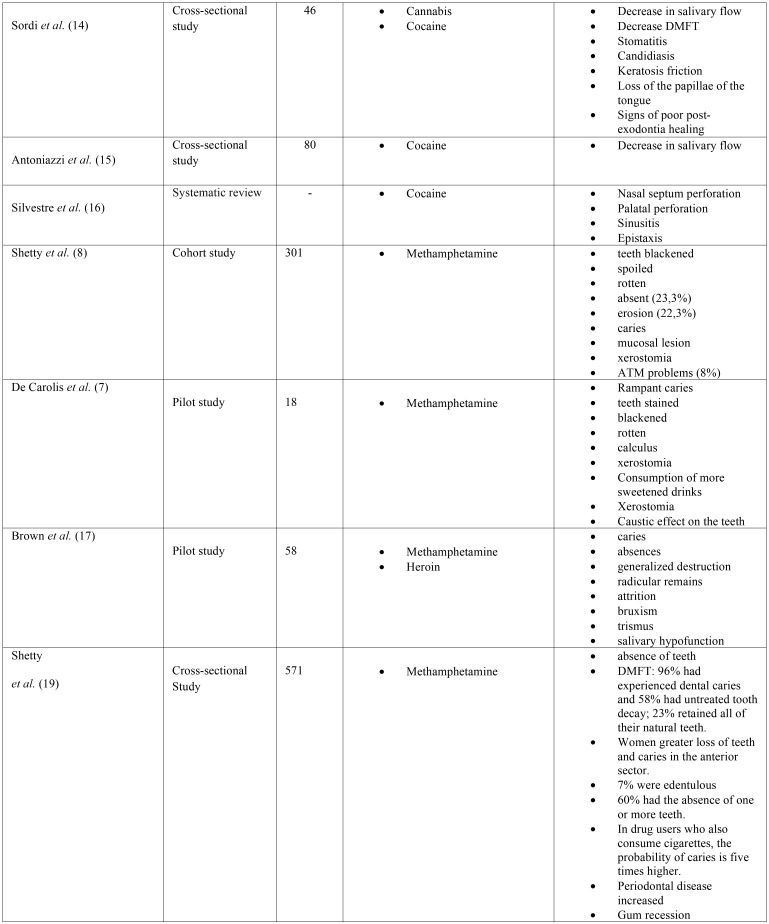
Description of the main characteristics of the selected studies.

**Table 1 cont.1:**
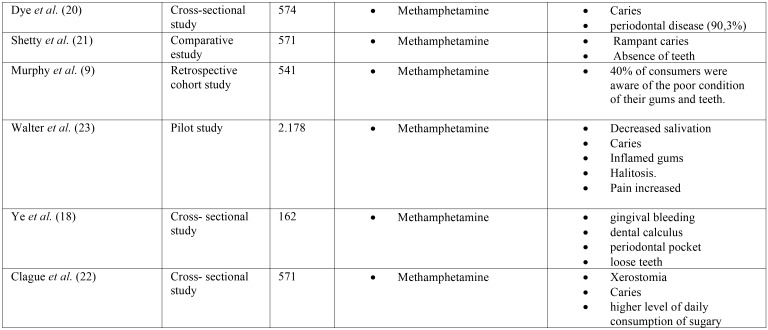
Description of the main characteristics of the selected studies.

## Discussion

It is difficult to identify and isolate the root causes of oral diseases among addicts, since they show a variety of unhealthy behaviors. Poor oral hygiene, increased sugar intake, and inappropriate nutrition, life style and limited education do not favor the proper use of dental service (2).

The authors showed the oral side effects of cannabis: periodontal disease, xerostomia, increased risk of decay, erosion of enamel due to cannabinoid hyperemesis, pulpitis, increased risk of candidiasis, leukoplakia, precancerous lesions and oral carcinoma (2,11).

Thomson *et al.* (12) observed that cannabis smoking was a risk factor for periodontal disease among young adults. One or more periodontal sites with a 4 mm or greater loss of clinical attachment level (CAL) reached 29.3% at 32 years of age.

Users with high exposure present 7 times greater the risk compared to none users, of developing periodontal disease. The periodontal effects of tobacco smoke are thought to occur via the systemic effects of nicotine and other toxic constituents on immune function and the inflammatory response within the periodontal tissues. Cannabis contains more than 400 compounds, the constituents are similar to tobacco those have been reported to carry systemic health risks and have histopathological effects that are similar to those of tobacco smoke (12).

Also the study by Gigena *et al.* (13) revealed that adolescent consumers of cannabis and cocaine have a higher risk of developing periodontal disease, only 20% of consumers had periodontal health.

In fact, the adolescent consumers had a higher plaque index than their peers who did not use drugs, people who are still cannabis smokers are characterized by lack of concern for personal hygiene and appearance, suggesting self-abandonment (13).

A study conducted by Mateos Moreno *et al.* (1) showed that the most frequent oral pathology was Periodontitis in 81% of the cases analyzed (1).

They (1) also recorded xerostomia in 64% of consuming patients. According to a study by Sordi *et al.* (14) they also observed a decrease in salivary flow, although they emphasize that determining the real cause of this disorder in this type of patients is complicated as they are polydrug users (1,14).

Mateos Moreno *et al.* (1) also detected a reduction in the buffer capacity of saliva in the drug users analyzed and an increase of *Streptococcus Mutans* and *Lactobacillus* in the saliva of individuals addicted to substances. This data does not comply with the results of Gigena *et al.* (13) who did not find xerostomia and any decrease in the buffer capacity in the sample of addicted individuals who were analyzed.

Saini *et al.* (11) noted an increased risk of caries in cannabis users, as well as a higher prevalence of dental erosion due to cannabinoid hyperemesis. Shekarchizadeh *et al.* (2) refered that cannabis itself is not the cause of an increase in the incidence of caries, even though the lifestyle of marijuana and hashish addicts leads to a decrease in salivation, thus favoring the increase of cavities. They also described the appearance of pulpitis during the period of cannabis use; the authors explain that this manifestation is probably due to an adverse effect of cannabis (2,11).

The authors Gigena *et al.* (13) analyzed the DMFT (Decayed, Missing, Filled, Teeth) index, comparing it with a sample of individuals who did not have any addiction to substances. The index DMFT was twice as high in the group of marijuana and cocaine addicts as in the group of individuals without toxic habits. More specifically, they observed that factor M (missing), was three times higher in the group of cannabis users with respect to the group of individuals who did not use drugs. Moreover, the results of authors Mateos Moreno *et al.* (1) coincide with those found in the study by Gigena *et al.* (13), demonstrating a prevalence of rampant caries in users of cannabis and other drugs, with 23.9% of radicular affectation, a higher DMFT value in the group substance abusers with respect to the control group that did not use any psychotropic substances.

The study of Sordi *et al.* (14) gave different results on the DMFT index. As analyzed by these authors, the index was lower in the group of cannabis and cocaine users than in the control group of individuals without any drug addiction, the prevalence of caries and missing teeth was higher in the group of cannabis and cocaine users but the number of filled teeth was higher in the control group. this could be a consequence of higher access to dental treatment by the control group (14).

They also observed a higher prevalence of lesions in the oral mucosa in individuals who used cannabis: stomatitis, candidiasis, keratosis by friction, loss of the papillae of the tongue and obvious signs of poor post-exodontia healing (14). Mateos Morenos *et al.* (1) detected the presence of candidiasis in 78.8% of the addicted individuals, 10.9% had angular cheilitis, 9.4% ulcers in the oral mucosa and 4.8% had leukoplakia and mucositis and 1.6% papillomas.

Saini *et al.* (11) and Shekarchizadeh *et al.* (2) reported that the use of cannabis can increase the risk factor for developing head and neck cancer.

Brand *et al.* (5) in their review say that cocaine users often suffer from bruxism and pain at the level of temporomandibular joint and masticatory muscles; it describe cases of gingival recessions, sometimes also with bone loss or erythematous and ulcerated gums in individuals who use cocaine orally. These patients all present a decreased salivary pH, thus increasing the risk of caries (5,11). Antoniazzi *et al.* (15) detected a decrease in salivary flow in consumers of crack (15). Cocaine can also generate involuntary jaw movements, these dyskinesias can be caused by both cocaine and crack (2). Among cocaine inhalers, very typical injuries were observed such as perforation of the nasal septum, palatal perforation, also chronic sinusitis, changes in the sense of smell and recurrent epistaxis, rhinolalia and regurgitation of solid food and liquids through the nares (2,5,11,16).

The perforations of the nasal septum are observed in 5% of cocaine users and may be due to the vasoconstrictor property of said drug, and to the substances that are added to cocaine, such as caffeine, talc, quinine and plaster which irritate the nasal mucosa. Another factor that may favor perforation of the septum nasal is a high presence of staphylococcus aureus in the nasal cavity (5).

Brown *et al.* (17) in their study confirm that the DMFT in heroin users studied was elevated, both by the presence of decay and absences. Individuals usually also have periodontal disease, inadequate oral hygiene habits and bad nutrition that worsens the oral situation. Due to the effects of the drug sometimes they do not perceive the sensation of pain that inflammation or infection can cause level of the oral cavity, ignoring the warning signs of the organism and therefore not seeking dental care (10,11,17).

Other complications that can be associated with heroin use are xerostomia, syndrome of the burning mouth, alteration of taste and difficulty in mastication. Additionally, many times pain management is not possible since analgesics and anesthetics do not have the desired effect in these patients (2,11).

Methamphetamine is of major importance as it is a very popular drug which causes a lot of oral destruction and diseases that sometimes oblige the patient to adopt the solution of a removable prosthesis due to the impossibility of saving the teeth remnants.

Shetty *et al.* (8) describe blackened, rotten and stained teeth, like the teeth of a “meth mouth”, that is, the conditions of the teeth of a person using MA (methamphetamine) (8).

In relation to this description of rampant caries for MA users is the study by De Carolis *et al.* (7), which adds to the description of “meth mouth”, teeth that are liable to fracture, and also another important feature: that the affectation of caries appears above all at the vestibular, cervical and proximal levels both in the maxilla and mandible with increasing involvement of the crown (7), until radicular remnants can appear as a consequence of the great coronal destruction (17,18).

The study conducted by Shetty *et al.* (8), looks for a relationship between the use of MA and the presence of an increase in dental diseases compared to those who do not use this type of drug. Among the participants of this study using the DMFT index, 23.3% had fractured or lost teeth, 22.3% had eroded or bruxism-related problems, and 8% had a problem at the temporomandibular joint (TMJ) level and, above all, pain associated with TMJ was detected in women (8).

Women, according to the study by Shetty *et al.* (19) had a higher number of tooth loss and decay, especially in the anterior sector (19).

According to Dye *et al.* (20) untreated caries occurred between 83-87% of adult consumers of MA and in men, and there were at least five areas affected by caries in the previous sector (94-97%) and a DMFT present in 93.3% of users (20).

The absence of one or more teeth was observed in 60% of MA consumers and due to this large percentage of dental absences, despite the young age (between 18 and 34 years old), 13.3% had a total or partial removable prosthesis. These data were collected among the consuming participants who preferably smoked MA (64.2%) (8).

The results of Shetty *et al.* (19) when comparing drug addicts with the healthy population, show that the dental diseases present in the first group was 41.3% with a 4.58% of tooth loss, with respect to the loss of the general population which was 1.96% (19). In addition, untreated caries amounted to 58% compared to 27% of the population (19).

MA consumers were twice as likely to have untreated caries and four times as likely to have cavities, while the possibility of having all teeth was 40% less for addicts than for non-addicts (21).

The caries present in the consumers of MA, were 80% in the molars and always in the posterior sector. In the anterior sector, the two central incisors have 20% more surfaces affected with caries with respect to the normal population; in fact the most affected teeth are the central incisors, followed by the premolars and molars (21).

The greatest lack of teeth of drug addicts is due to the fact that for financial reasons, they had to extract their teeth instead of treating them, especially in the posterior sector because the cost increases when there is a great destruction of the crown.

According to studies by Clague *et al.* (22), people who use MA tend to drink more sugary drinks. This habit, due to poor oral hygiene, means that they present more caries (22).

Another possible factor that leads to a poor oral state of MA users is the xerostomia that comes from the alteration caused by MA at the physiological level (7,9,18,22).

In addition, the inhalation of MA can cause a caustic effect on the teeth and this may be due to the excretion of the drug through the crevicular fluid (7).

Another habit that drug addicts tend to have is the consumption of cigarettes; in fact, there is a major difference between the teeth present in the mouth of consumers of MA and smokers, with respect to those who do not smoke - being 41.3% and 17.5% respectively (19).

The periodontium is affected by the consumption of MA and the levels of periodontitis were 89% among those who also smoke cigarettes, while 75% of those who do not smoke did not have periodontitis. Among cigarette consumers, there was more dental recession compared to non-smokers. Therefore, in their opinion, there is a definite relationship between the use of cigarettes and caries (almost three times more compared to the non-smoker) and this is affirmed as a risk factor that leads to the greater presence of untreated caries and root caries, but it is still unknown how it can be related to periodontitis (17,18).

In the article by Walter *et al.* (23) for the first time it is also affirmed that not only is there affectation at the level of salivation, teeth and gums, but also the presence of bad breath (23).

In the study by Brown *et al.* (17), one third of the MA drug addicts analyzed reported a “cotton mouth” sensation with the need to drink while eating in order to be able to swallow (17).

With every year that passes, MA users increase the dry mouth feeling by 3% (9).

After carrying out this review, we can conclude that:

- The most commonly used drugs are methamphetamine, heroin and cocaine in its different forms of administration: injected, smoked and inhaled, and smoked cannabis. Bearing in mind that, in addition, many drug users are addicted to several of these substances.

- In the oral cavity, the effects of the different drugs are similar, producing rampant caries, periodontal disease and xerostomia, thus raising the DMFT indices and candida infection. Methamphetamine, on the other hand, produces a very characteristic mouth, the so-called “meth mouth”. Cannabis produces dental erosion due to cannabinoid hyperemesis; cocaine has also been related to TMJ alterations and pain, and in its inhaled form it can produce palatal perforation; heroin, in turn, produces dysgeusia and alterations in chewing.

- The role of the dentist is aimed at detecting, in the first place, this type of patient, preventing the complications derived from the consumption of drugs, as well as the bad health habits they present and consequently treating the complications. Likewise, it is necessary to insist on the cessation of the consumption once the dental treatments have been carried out.
